# OUR WAYS ARE A GIFT: NATIVE WAYS OF LIFE AND SPIRITUALITY AMONG ALASKA NATIVE ELDERS

**DOI:** 10.1093/geroni/igae098.1125

**Published:** 2024-12-31

**Authors:** Lena Thompson, Steffi Kim, Jordan Lewis

**Affiliations:** University of Minnesota Duluth, Duluth, Minnesota, United States; University of Alaska, Anchorage, Alaska, United States; University of Alaska Fairbanks, College of Indigenous Studies, Fairbanks, Alaska, United States

## Abstract

Spirituality plays an important role and tends to increase in importance as one ages, including for Alaska Natives. Spirituality is also one of the guiding principles of aging well and contributes to other aspects of Elders’ lives. The presence of spirituality does not ensure successful aging, but rather enables them to maintain a positive and healthy life. This presentation is part of a parent study that consists of 162 qualitative interviews with AN Elders over the course of 16 years, representing over 21 rural and urban communities across Alaska, and were analyzed using thematic analysis to explore the role of spirituality as a part of Native ways of life and successful aging. The Elders in this study practiced different forms of spirituality, including prayer, spending time on the land, participating in their Native arts and crafts, and spending time with family and community. Elders discussed how spirituality is found within most of their Native ways of life, including hunting, gathering, and preparing Native foods, and spending time with family and community. Spirituality is self-defined, unlike religion which relates to participation in a Western form of religion, like attending church. Some Elders in this study are both spiritual and religious, so it is important to ask about these practices and understand how they impact their understanding of aging in a good way. This presentation will conclude with a discussion of recommendations families, communities, and programs can do to integrate spiritual and religious activities into their programs to promote successful aging.

